# The Association of Seasonal Variations and COVID-19 Clinical Features: A Comparative Study on the Fourth and Fifth Waves

**DOI:** 10.1155/2022/8347103

**Published:** 2022-08-27

**Authors:** Kasra Karvandian, Kiana Tadbir Vajargah, Shabnam Beigi, Narjes Mohammadzadeh, Mohammad Ashouri, Shahram Samadi, Mohsen Zamani

**Affiliations:** ^1^Department of Anesthesiology and Intensive Care, Imam Khomeini Hospital Complex, Tehran University of Medical Sciences, Tehran, Iran; ^2^School of Medicine, Tehran University of Medical Sciences, Tehran, Iran; ^3^Department of Surgery, Imam Khomeini Hospital Complex, Tehran University of Medical Sciences, Tehran, Iran

## Abstract

**Purpose:**

The COVID-19 pandemic has overwhelmed many healthcare systems. Seasonality is a feature of several infectious diseases. Studies regarding the association of seasonal variations and COVID-19 have shown controversial results. Therefore, we aimed to compare COVID-19 characteristics and survival outcomes between the fourth and fifth waves in Iran, which corresponded to spring and summer, respectively.

**Methods:**

This is a retrospective study on the fourth and fifth COVID-19 waves in Iran. One hundred patients from the fourth and 90 patients from the fifth wave were included. Data from the baseline and demographic characteristics, clinical, radiological, and laboratory findings, and hospital outcomes were compared between the fourth and fifth COVID-19 waves in hospitalized patients in Imam Khomeini Hospital Complex, Tehran, Iran.

**Results:**

The fifth wave patients were more likely to present with gastrointestinal symptoms than the patients from the fourth wave. Moreover, patients in the fifth wave had lower arterial oxygen saturation on admission (88% vs. 90%; *P* = 0.026), lower levels of WBCs (neutrophils and lymphocytes) (6300.00 vs. 8000.00; *P* = 0.004), and higher percentages of pulmonary involvement in the chest CT scans (50% vs. 40%; *P* < 0.001). Furthermore, these patients had longer hospital stays than their fourth-wave counterparts (7.00 vs. 5.00; *P* < 0.001).

**Conclusions:**

Our study indicated that patients in the summer COVID-19 wave were more likely to present with gastrointestinal symptoms. They also experienced a more severe disease in terms of peripheral capillary oxygen saturation, percentages of pulmonary involvement in CT scans, and length of hospital stay.

## 1. Introduction

COVID-19 has emerged as a pandemic since its first reports in late December 2019 [1]. The disease caused by severe acute respiratory syndrome coronavirus 2 (SARS-CoV-2) has overwhelmed many healthcare systems worldwide [2]. According to the World Health Organization (WHO), a total of 380 million COVID-19 cases have been reported worldwide, including 5,680,741 deaths up to February 3^rd^, 2022. In the same period, there have been over 6.4 million confirmed cases of COVID-19 in Iran, including 132,000 reported deaths [3].

Seasonality is described as the changes, which occur within a year and are repeated across years [4]. Seasonality has been a feature of several infectious diseases, either acute or chronic [5], including influenza, RSV (respiratory syncytial virus), and MERS-CoV (Middle East respiratory syndrome coronavirus) infections [5]. Studies regarding the association of meteorological parameters and COVID-19 have provided controversial results [6–9]. Meteorological parameters, including temperature [10–16], relative humidity [12–17], average daylight hours [10], rainfall [13], and wind speed [11, 16], can potentially affect COVID-19 transmission, contributing to its fast spread across many countries. On the other hand, studies have shown heterogeneous and contradictory results, some of which do not confirm the association of meteorological factors with COVID-19 transmissibility [18, 19].

To the best of our knowledge, this is the first study on the Iranian population to explore the seasonal patterns of COVID-19 symptoms and severity. We aimed to compare COVID-19 characteristics between the fourth and fifth pandemic waves (corresponding to spring and summer, respectively). Thus, we investigated the association of COVID-19 clinical, laboratory, and radiological characteristics and outcomes with the seasonal changes in an observational study on the Iranian population.

## 2. Materials and Methods

This is a retrospective observational study. We used the data from two timeframes of March and June 2021, corresponding to the fourth and fifth COVID-19 waves in Iran, respectively. We selected one hundred ninety adult patients (100 and 90 from the fourth and fifth waves, respectively), aged over 18 years with available medical records, who were admitted to Imam Khomeini Hospital Complex during the study timeframe by a simple random sampling method from all COVID-19 patients. Patients undergoing outpatient management or patients with a hospital stay shorter than 24 hours or with incomplete medical records were excluded from the study. The protocol of this study was reviewed and approved by the Ethics Committee of the Tehran University of Medical Sciences (Registration no. IR.TUMS.IKHC.REC.1400.395). A code was assigned to each participant, and all data analyses were performed anonymously.

Data regarding baseline and demographic characteristics, clinical characteristics, radiological and laboratory findings, and hospital outcomes were compared between the fourth and fifth COVID-19 waves in hospitalized patients in Imam Khomeini Hospital Complex, Tehran, Iran.

### 2.1. Confirmation of Diagnosis

The COVID-19 diagnosis was confirmed based on definite evidence of COVID-19 pulmonary involvement in a spiral chest computed tomography (CT) scan or positive reverse transcription polymerase chain reaction (RT-PCR) conducted on pharyngeal swabs.

### 2.2. Baseline and Demographic Characteristics

Data regarding age, gender, smoking history, previous COVID-19 infection, and comorbidities were collected from the medical records. Comorbidities included diabetes mellitus (DM), hypertension (HTN), ischemic heart disease (IHD), chronic kidney disease (CKD), hepatobiliary disease, asthma, malignancy, and hypothyroidism.

### 2.3. Clinical Characteristics

We collected the information regarding COVID-19 signs and symptoms from patients' medical records. They included fever, shivering, cough, dyspnea, abdominal pain, myalgia, weakness, headache, nausea, vomiting, diarrhea, constipation, anosmia/ageusia, anorexia, conjunctivitis, oral manifestations (including ulcers and aphthous lesions), and peripheral capillary oxygen saturation (SpO2) on admission.

### 2.4. Radiological and Laboratory Findings

We cited radiologists' reports of pulmonary involvement (the presence and pattern of involvement, including ground-glass opacity (GGO) or consolidation, and the quantity of involvement reported as a percentage) from spiral CT scans of the chest. Laboratory findings such as complete blood count (CBC) with differentiation were retrieved from patients' medical records.

### 2.5. Hospital Outcomes

Hospitalization-related data, including the mode of respiratory support, length of hospital stay, and in-hospital deaths, were collected from the medical records.

### 2.6. Statistical Analysis

We carried out the Kolmogorov–Smirnov test to explore whether the data distribution was normal. We reported the mean ± standard deviation (SD) for normally distributed data and the median (interquartile range) for non-normally distributed data. We performed the independent sample *T*-test to compare quantitative variables, the Chi-square test to compare qualitative variables, and the Mann–Whitney *U* test to compare the non-normally distributed data between the two groups. All statistical analyses were conducted by the Statistical Package for Social Sciences software (SPSS Inc. Version 26). *P* values less than 0.05 were considered statistically significant.

## 3. Results

A total of 190 patients (100 from the fourth and 90 from the fifth COVID-19 waves) were included in the study. The overall characteristics of study participants are shown in Table 1.  Table 2 summarizes the baseline characteristics of the study participants. Hospitalized COVID-19 patients in the fifth wave had a significantly higher prevalence of smoking than those in the fourth wave. In contrast, the two waves did not differ significantly regarding the mean, age, and gender distribution of patients. In addition, patients' other comorbidities and their prevalence were not significantly different between the two waves.

Table 3 describes the clinical, laboratory, and radiological characteristics of patients in the fourth and fifth waves of COVID-19. Patients were more likely to experience cough and gastrointestinal symptoms, including nausea, vomiting, diarrhea, and constipation, during the fifth wave. Patients in the fifth wave presented with a more extensive pulmonary involvement in the spiral CT scan than those in the fourth wave (*U* = 5867, *P* < 0.001). Furthermore, the pattern of radiological findings was different between the two waves (*U* = 3965, *P* = 0.002). In addition, the Mann–Whitney *U* test showed a significantly lower SpO2 at the time of admission in patients in the fifth wave, when the prominent COVID-19 variant was the Delta variant (*U* = 3662, *P* = 0.026).

Table 4 illustrates the hospital outcomes of COVID-19 patients in the fourth and fifth waves. The Mann–Whitney test showed that the mode of respiratory support significantly differed between the fourth and fifth waves (*U* = 6148.5, *P* < 0.001). Although patients in the fifth wave required a longer hospital stay (7.00 (6) vs. 5.00 (3), *P* < 0.001), they were not significantly different from the patients in the fourth wave regarding in-hospital deaths.

## 4. Discussion

This study, to the best of our knowledge, is the first study on the Iranian population aiming at the comparison of COVID-19 characteristics between the fourth and fifth waves (corresponding to spring and summer 2021, respectively). We investigated the potential association of COVID-19 clinical, laboratory, and radiological features and outcomes with seasonal changes.

Our results indicate that patients in the fifth wave (corresponding to the Delta variant of COVID-19) had a lower arterial oxygen saturation on admission, lower levels of WBCs (neutrophils and lymphocytes), and higher percentages of pulmonary involvement in the chest CT scans. Moreover, these patients had longer hospital stays than their fourth-wave counterparts. In other words, Iran's summer wave (fifth wave) had a more severe clinical phenotype with a more severe pulmonary involvement. Since comorbidities were comparable between the two waves, the more severe clinical feature in the fifth wave, in part, can be explained due to the dominant Delta variant and the potential disease seasonality. A study has shown that the Delta variant is highly transmissible, accounting for potentially more severe diseases [20]. In contrast, other studies have not confirmed this increase in disease severity during Delta-predominant waves [21–23]. Patients in the fifth wave were more likely to be managed by noninvasive ventilation and less likely to be intubated despite the more severe disease and pulmonary involvement. We hypothesize that higher experience in COVID-19 management could have led to a higher threshold for endotracheal intubation in the fifth COVID-19 wave in Iran.

Several studies have compared COVID-19 waves worldwide from the first wave till now. These comparative studies have yielded inconsistent results, even within one country [24]. In a study conducted in the United States, new COVID-19 cases were positively linked with temperature and humidity [25]. A modeling study in the United States suggested that the increase in temperature and humidity decreases COVID-19 spread [26]. In other studies, researchers have suggested that the rise in temperature decreases the risk of COVID-19, but higher levels of humidity increase the disease risk [27, 28]. Studies from Italy have compared the clinical features and outcomes of the first and second COVID-19 waves. Despite some conflicting results, their results showed that the second-wave patients experienced a less severe disease with better outcomes [29, 30]. In a cohort study conducted in South Africa, the second wave was associated with higher hospital admissions and increased mortality than the first wave [31]. In studies performed in India, the patients in the second wave had more comorbidities [32] and experienced a greater severity of disease [33], and their mortality was significantly higher than their first-wave counterparts [32, 34]. A comparative study on the first and second COVID-19 waves in southern Germany showed improved survival outcomes in the second wave. This survival improvement was mainly observed among patients requiring intensive care and mechanical ventilation. It corresponded to the more frequent use of nasal high-flow (NHF) oxygen and noninvasive ventilation (NIV) instead of intubations [35]. Another study from Germany indicated that although in-hospital mortality did not differ between the first and second waves, patients in the second wave were more likely to be treated as outpatients and had a significantly shorter duration of hospitalization [36]. These inconsistent findings highlight the importance of country-specific and region-specific data when comparing the COVID-19 waves.

The fourth and fifth COVID-19 waves in Iran corresponded to spring and summer, respectively. Our results revealed that in the fifth wave, which occurred in the summer, gastrointestinal symptoms were the predominant feature of COVID-19. Accordingly, it is implied that the pattern of COVID-19 presentation may differ across seasons and probably regions and countries [37]. Therefore, we believe that implementing identical diagnostic criteria across different seasons and geographical locations appears inaccurate. Such potentially insensitive criteria can lead to underdiagnoses and unsuccessful break of the chain of transmission during the COVID-19 pandemic. Our findings suggest that the clinical criteria for COVID-19 diagnosis should be modified according to seasons and locations. This spatiotemporal approach to altering the diagnostic criteria would contribute to timely case findings, followed by appropriate restrictive measures.

Diagnostic testing for COVID-19 is crucial in the prevention and control strategy [38]. To achieve this goal, we suggest that patients with nonspecific COVID-19 symptoms, considering seasonal variabilities, undergo screening tests using rapid antigen testing in the first place. WHO recommends rapid antigen testing as a screening tool for COVID-19 in individuals who have been in contact with COVID-19 patients, primarily those with an increased risk of developing severe disease or those with high levels of exposure [38, 39]. According to the WHO's interim guidance on ‘Antigen-detection in the diagnosis of SARS-CoV-2 infection,' updated in October 2021, these tests are more reliable in regions with ongoing community transmission, defined as a test positivity rate of at least 5% [38]. Rapid antigen tests provide results in several minutes, which is significantly shorter than RT-PCR [38, 40]. This shorter interval is critical because a vital driver of viral spread is presymptomatic or paucisymptomatic transmission [41, 42]. Therefore, rapid antigen tests provide the opportunity for timely diagnosis and interruption of disease transmission [38]. If rapid antigen tests are positive, patients will undergo the more accurate but time-consuming diagnostic testing, i.e., RT-PCR. In case of positive RT-PCR, these patients will undergo containment measures, including quarantine and close contact testing, to prevent viral spread.

By February 2022, Iran has entered the sixth COVID-19 peak, when the Omicron variant is dominant. Keeping in mind that this peak will possibly become established in the winter, we suggest that further investigations be made regarding the pattern of COVID-19 presentations. The findings of this study and the data from the potential winter peak can be used to provide a more comprehensive comparison of COVID-19 manifestations across different seasons.

Our study had several limitations. First, it was an observational, retrospective study with a modest sample size conducted in one hospital. Although our hospital is a tertiary referral center where patients are admitted from all regions of Iran, the single-center nature of our study could potentially limit the generalizability of our findings. Second, whether the Delta variant of SARS-CoV-2 was responsible for the disease severity in the fifth wave was not documented by diagnostic tests of variances in our hospital because these tests were not conducted for all patients at our hospital. Therefore, the predominance of this variant in our hospital was implied by the predominance of the Delta variant in Iran's fifth wave [43].

## 5. Conclusions

In conclusion, we observed that patients in the fifth COVID-19 wave, which corresponded to summer in Iran, were more likely to present with gastrointestinal symptoms. In addition, they had lower oxygen saturation on admission and higher percentages of pulmonary involvement in CT scans. Despite the longer duration of hospitalization in the fifth wave, patients did not differ significantly regarding in-hospital mortality compared to patients in the fourth wave. Based on our findings, we suggest that the clinical diagnostic criteria for COVID-19, even in the same region and hospital, should be dynamic, corresponding to the seasons and times of the year.

## Figures and Tables

**Table 1:** Characteristics of the study participants (*N* = 190).

Characteristics	Value^*∗*^
Age (years)	54.98 ± 14.36
Female gender	74 (38.9%)
Past medical history	
Smoking	32 (16.8%)
DM	55 (28.9%)
HTN	62 (32.6%)
IHD	25 (13.2%)
CKD	12 (6.3%)
Hepatobiliary diseases	11 (5.8%)
Asthma	11 (5.8%)
Malignancy	4 (2.1%)
Hypothyroidism	16 (8.4%)
Clinical manifestations	
Fever	123 (64.7%)
Shivering	82 (43.2%)
Cough	157 (82.6%)
Dyspnea	150 (78.9%)
Abdominal pain	18 (9.5%)
Myalgia	119 (62.6%)
Weakness	96 (50.5%)
Headache	54 (28.4%)
Nausea/vomiting	21 (11.1%)
Diarrhea	17 (8.9%)
Constipation	15 (7.9%)
Anosmia/ageusia	28 (14.7%)
Anorexia	74 (38.9%)
Conjunctivitis	13 (6.8%)
Oral manifestations	16 (8.4%)
SpO_2_ on admission (percent)	88 (6)
Radiological and laboratory characteristics	
Chest CT involvement	
(i) Percentage	40 (20)
(ii) Type	
No involvement	3 (1.6%)
GGO	176 (92.6%)
Consolidation	11 (5.8%)
WBC count (cell/microliter)	6950.00 (4375)
Neutrophils (cell/microliter)	5838.00 (4153)
Lymphocytes (cell/microliter)	807.50 (591)
Mode of respiratory support	
(i) Room air	8 (4.2%)
(ii) Nasal cannula	12 (6.3%)
(iii) Simple mask	25 (13.2%)
(iv) Reservoir mask	130 (68.4%)
(v) NIV	8 (4.2%)
(vi) Intubation	7 (3.7%)
(vii) Tracheostomy	0 (0.0%)
Hospitalization period (days)	6.00 (3)
In-hospital deaths	13 (6.8%)

^*∗*^Values are reported as mean ± SD, median (IQR), or number (percentages). NIV: noninvasive ventilation.

**Table 2:** Baseline characteristics of patients in the 4^th^ and 5^th^ waves of COVID-19^*∗*^.

Characteristics	4^th^ wave (*n* = 100)^*∗*^	5^th^ wave (*n* = 90)^*∗*^	*P* value^*∗∗*^
Age (years)	55.73 ± 15.54	54.14 ± 12.95	0.449
Female gender	44 (44.0%)	30 (33.3%)	0.140
Smoking	10 (10.0%)	22 (24.4%)	0.011
DM	34 (34.0%)	21 (23.3%)	0.112
HTN	32 (32.0%)	30 (33.3%)	0.878
IHD	9 (9.0%)	16 (17.8%)	0.087
CKD	4 (4.0%)	8 (8.9%)	0.234
Hepatobiliary diseases	6 (6.0%)	5 (5.6%)	1.000
Asthma	6 (6.0%)	5 (5.6%)	1.000
Malignancy	2 (2.0%)	2 (2.2%)	1.000
Hypothyroidism	6 (6.0%)	10 (11.1%)	0.296

^*∗*^ Values are reported as mean ± SD or number (percentages). ^*∗∗*^ Obtained from the chi-square test and the independent sample *T*-test, where appropriate. DM: diabetes mellitus; HTN: hypertension; IHD: ischemic heart disease; CKD: chronic kidney disease.

**Table 3:** Patients' clinical, laboratory, and radiological characteristics of 4^th^ and 5^th^ COVID-19 waves^*∗*^.

Manifestations and findings	4^th^ wave (*n* = 100)^*∗*^	5^th^ wave (*n* = 90)^*∗*^	*P* value^*∗∗*^
Fever	62 (62.0%)	61 (67.8%)	0.449
Shivering	39 (39.0%)	43 (47.8%)	0.243
Cough	75 (75.0%)	82 (91.1%)	0.004
Dyspnea	75 (75.0%)	75 (83.3%)	0.212
Abdominal pain	11 (11.0%)	7 (7.8%)	0.471
Myalgia	60 (60.0%)	59 (65.6%)	0.456
Weakness	50 (50.0%)	46 (51.1%)	0.886
Headache	28 (28.0%)	26 (28.9%)	1.000
Nausea/vomiting	2 (2.0%)	19 (21.1%)	<0.001
Diarrhea	1 (1.0%)	16 (17.8%)	<0.001
Constipation	3 (3.0%)	12 (13.3%)	0.013
Anosmia/ageusia	13 (13.0%)	15 (16.7%)	0.541
Anorexia	39 (39.0%)	35 (38.9%)	1.000
Conjunctivitis	5 (5.0%)	8 (8.9%)	0.390
Oral manifestations	6 (6.0%)	10 (11.1%)	0.296
SpO_2_ on admission (percent)	90.00 (6)	88 (6)	0.026
Chest CT involvement			
(i) Percentage	40.00 (20)	50.00 (20)	<0.001
(ii) Type			0.002
No involvement	0.00	3.33	
GGO	90.00	95.56	
Consolidation	10.00	1.11	
WBC count (cell/microliter)	8000.00 (4825)	6300.00 (3675)	0.004
Neutrophils (cell/microliter)	6409.00 (4702)	5020.00 (3333)	0.016
Lymphocytes (cell/microliter)	908.50 (755)	763.50 (473)	0.027

^*∗*^Values are reported as median (IQR) or number (percentages). ^*∗∗*^Obtained from the chi-square test and the Mann–Whitney test, where appropriate. SpO_2_: peripheral capillary oxygen saturation; WBC: white blood cell.

**Table 4:** Hospital outcomes of patients in the fourth and fifth COVID-19 waves^*∗*^.

Characteristics	4^th^ wave (*n* = 100)^*∗*^	5^th^ wave (*n* = 90)^*∗*^	*P* value^*∗∗*^
Mode of respiratory support			
(i) Room air	8 (8.0%)	0 (0.0%)	<0.001
(ii) Nasal cannula	10 (10.0%)	2 (2.2%)	
(iii) Simple mask	25 (25.0%)	0 (0.0%)	
(iv) Reservoir mask	49 (49.0%)	81 (90.0%)	
(v) NIV	2 (2.0%)	6 (6.7%)	
(vi) Intubation	6 (6.0%)	1 (1.1%)	
(vii) Tracheostomy	0 (0.0%)	0 (0.0%)	
Hospitalization period (days)	5.00 (3)	7.00 (6)	<0.001
In-hospital deaths	8 (8.0%)	5 (5.6%)	0.575

^*∗*^Values are reported as median (IQR) or number (percentages). ^*∗∗*^Obtained from the Chi-square test, Fischer's exact test, and the Mann–Whitney test, where appropriate. NIV: noninvasive ventilation.

## Data Availability

The datasets generated or analyzed during the current study are not publicly available but are available from the corresponding author on reasonable request.

## Abbreviations

COVID-19: Coronavirus disease 2019
SARS-CoV-2: Severe acute respiratory syndrome coronavirus 2
CT: Computed tomography
RT-PCR: Reverse transcription polymerase chain reaction
DM: Diabetes mellitus
SpO_2_: Peripheral capillary oxygen saturation
GGO: Ground-glass opacity
WBC: White blood cell
CBC: Complete blood count
HTN: Hypertension
IHD: Ischemic heart disease
CKD: Chronic kidney disease
IQR: Interquartile range
SD: Standard deviation
NIV: Noninvasive ventilation.
